# Physical Violence and Associated Factors among Women of Reproductive Age in Gedeo Zone, Southern Ethiopia

**DOI:** 10.4314/ejhs.v31i5.6

**Published:** 2021-09

**Authors:** Zemenu Yohannes Kassa, Abebaw Abeje, Tebeje Ashegu, Nebiha Hadra

**Affiliations:** 1 Department of Midwifery College of Medicine and Health Sciences, Hawassa University

**Keywords:** Physical violence, Reproductive age women, Ethiopia

## Abstract

**Background:**

Physical violence against women of reproductive age is a significant public health problem worldwide. This study aimed to assess physical violence and associated factors among women of reproductive age.

**Method:**

A community-based cross-sectional study design was implemented from August 1 to September 30, 2018, including women of reproductive age in Gedeo Zone Southern Ethiopia. A stratified, two-stage cluster sampling technique was used. Finally, the study population was selected from the respective source population using a simple random sampling technique. Data were checked, coded, and entered Epi data version 3.1 and exported to SPSS version 20 for analysis. The wealth index was computed using the principal component analysis. Bivariate and multivariable analyses were computed to identify the determinants of physical violence among women of reproductive age.

**Results:**

Experiencing at least one type of physical violence among women of reproductive age was 14.7% (95%CI: 11.7, 17.4). Study participants whose spouse had any habit (AOR: 3.56; 95%CI: 1.75, 7.25) and whose spouse had watched pornography counterpart ((AOR: 1.58; 95%CI: 1.02, 3.17) had significantly higher odds of experiencing physical violence among women of reproductive age. Spouses had any habit like alcohol drinking, chat chewing, cigarette smoking, and seeing pornography significantly increased physical violence among reproductive-age women. Therefore, the responsible stakeholders should work on the means to the spouse can alleviate any form of habit like alcohol drinking, chat chewing, cigarette smoking, and seeing pornography could decrease physical violence in women of reproductive age.

## Introduction

Violence against women is a reproductive health problem in all societies regardless of culture, ethnicity, and socio-economic status (1). It is the most shameful human rights violation, and it has no boundaries of geography, culture, or wealth (2). In worldwide, one in three women have experienced gender-based violence and are among the foremost causes of death and disability. Gender-based violence is a significant obstacle to achieving stakeholders' sustainable development goals 3 (3, 4).

Physical violence against women is also a significant public health concern that has reached endemic proportions, as well as a fundamental violation of women's rights. Violence against women is actual or threatened physical, sexual, psychological, and emotional abuse based on gender. Women of reproductive age have experienced physical injury and chronic health problems like depression and anxiety. These problems go to pregnancy due to a social problem that devastates maternal and neonatal health during pregnancy and childbirth (5,6). The women also have trouble using family planning effectively, have experienced an unintended pregnancy, unsafe abortion, have contracted STI, depression and substance abuse (7). A study showed that 28.6% of women of reproductive age experienced physical violence in Zimbabwe (8). Additionally, a study showed that in Egypt, 22.4% of women of reproductive age experienced physical violence (9). In Ethiopia, 58.4% of married women of reproductive age experienced physical violence (10).

Abuse during pregnancy has been associated with poor maternal physical health outcomes such as increased STIs, preterm labour, vaginal bleeding, placental abruption, cesarean delivery, hemorrhage and infection (11). Gender-based violence has caused adverse birth outcomes for the fetus, and neonates were found to be associated with low-birthweight and neonatal death (12, 13, 14). Physical violence during pregnancy is harmful to both and mother and the unborn baby (1). Worldwide 42% of women have experienced physical and sexual violence by a partner, which results in injuries (15).

Women experiencing IPV during pregnancy have a high rate of miscarriage, more complications during pregnancy, sexually transmitted infections (including HIV), and a higher prevalence of mental disorders such as depression, anxiety, sleep, and eating disorders occurred (16–19).

Little attention has been given to understanding physical violence on women of reproductive age, which forward further explanation. This study aims to assess the prevalence of physical violence and associated factors, which directly impact maternal and neonatal health at large family health. Stakeholders to achieve the Sustainable Development Goal (SDG) on maternal, neonatal, and child health, alleviation of gender-based violence is crucial. This finding will input stakeholders, health care providers, policymakers, community, NGO, and health administration.

## Methods and Materials

**Study setting, study design and period**: A community-based cross-sectional study design was implemented from August 1 to September 30, 2018, including women of reproductive age in Gedeo Zone Southern Ethiopia. Gedeo is one of the zones in Southern Nations, Nationalities and Peoples Regional State (SNNPRS). It is a home of 8 (2 towns and six rural) woredas and 148 kebeles (13 towns and 135 rurals). Its administrative city is Dilla which is 377km South of Addis Ababa, the capital city of Ethiopia. According to the 2007 Census conducted by the Ethiopian Central Statistical Agency, the zone has a total population of 847,434 and a population density of 699.84. A total of 179,677 households were counted in this zone. According to the zonal health office, the current (2016/2017) estimated total population is 1,112,951, of which 239,053 are women of reproductive age (15–49).

**Study population**: All women in women of reproductive age in this study were a source of population. All randomly selected women of reproductive age were included in this study. Women who had difficulty communicating during the study period, like critically ill and psychiatric patients, were excluded.

**Sample size determination**: The sample size was calculated using a single population proportion formula similar to physical violence in women of reproductive age in Northwest Ethiopia was (58.4%) (10). The study's significance level is 5 %(a=0.05), the margin of error 5 % (d=0.05), and the non-responsive rate of 5%; the study is multistage sampling, and in this study, 1.5 design effect was used. The total sample was 588.

**Sampling technique and sampling procedures**: A stratified, two-stage cluster sampling technique was used. Initially, all administrative kebeles in the Gedeo Zone were stratified into town and rural. Then two urban and 21 rural kebeles were randomly selected. Census was conducted in each randomly selected kebele to identify women of reproductive age. The sample size was proportionally allocated for each selected kebele depending on the number of women of reproductive age. Finally, the study population was selected from the respective source population using a simple random sampling technique (computer-generated random numbers were used).

**Operational defining**: Physical violence is any acts like slapping, firing, kicking, pushing, sticking, gunshot, and burning (20).

**Outcome variable**: At least one form of physical violence were the outcome variable for this study. Types of physical violence were slapping, sticking, pushing, throwing, weapon, burning, and firing. Therefore, physical violence experienced said to be if and only if any domestic violence experiences it. Information about this domestic violence is secured based on women of reproductive age self-report.

**Data collection procedure and quality control**: The preliminary survey/census of women of reproductive age in the selected kebeles was carried out before the actual data collection. The data were collected using structured and pretested interview questionnaires from home to home. The questionnaires were prepared for reviewing different kinds of literature, standard demographic, and health survey (DHS) questionnaires. First, the questionnaires were written in English and then translated to a local language, Gedeo'ffa, back to English to check the consistency. The questionnaire has consisted of sociodemographic and physical violence. The questionnaires were pretested 5% at another kebele having similar socio-cultural characteristics with the study subject. The tool was checked reliability during the pretest, and the alpha coefficient was calculated at 0.78. A total of 10 trained data collectors who completed grade 10 or 12 and were proficient in Gedeo'ffa and two supervisors with Master of Public Health (MPH) were recruited for data collection. Throughout the data collection, data collectors were supervised, regular meetings were held among the data collectors, supervisors, and investigators to raise, discuss and solve problematic issues. Two more additional visits were made for participants who were not available during the first visit. The collected data were reviewed and checked for completeness before data entry.

**Data management**: Data were checked, coded, and entered Epi data version 3.1 and exported to SPSS version 20 for analysis. The wealth index was computed using the principal component analysis. Descriptive statistics was employed to display the study findings. Bivariate and multivariable analyses were computed to identify the determinants of physical violence in women of reproductive age. All explanatory variables with a P-value of less than 0.2 in the bivariate analysis were included in the multivariable analysis. Finally, statistical significance was considered at a P-value less than 0.05.

## Results

**Socio-demographic characteristics**: More than half (56.7%) participants were in the age groups 25–34 years, 40.5% of participants cannot read and write, and 51.2% were housewives (Table 1).

**Table 1:** Socio-Demographic characteristics in women of reproductive age in Gedeo zone, 2018

Variable		Number(n=580)	Percent
Age	15–25	67	11.6
	25–34	329	56.7
	35–44	161	27.8
	45–49	23	4.0
Ethnic	Gedeo	470	81.0
	Oromo	42	7.2
	Amhara	36	6.2
	Others ^a^	18	3.1
	Sidama	14	2.4
Educational status of participants	Cannot read and write	235	40.5
	Can read and write	57	9.8
	Primary school	193	33.3
	Secondary school	60	10.3
	College and above	35	6.0
Occupation of participants	Housewife	297	51.2
	Merchant	180	31.0
	Government employee	39	6.7
	Farmer	50	8.6
	Others*	14	2.4
Residence	Rural	361	62.2
	Urban	219	37.8
Marital status of participants	Married	554	95.5
	Divorced	17	2.9
	Widowed	9	1.6
Husband's educational status	Cannot read and write	78	13.4
	Can read and write	49	8.4
	Primary school	232	40.0
	Secondary	132	22.8
	College and above	89	15.3
Husband's occupation	Farmer	272	46.9
	Merchant	158	27.2
	Government employee	107	18.4
	Daily laborer	35	6.0
	Others@	8	1.4
Wealth index	Poor	205	35.3
	Middle	175	30.2
	Rich	200	34.5
Mobile	Yes	217	37.4
	No	363	62.6
Radio	Yes	219	37.8
	No	361	62.2

Others a= Guragie and silty, others*=daily labor, Others@=pension

Physical violence among the reproductive age group: Experiencing at least one form of physical violence such as slapping, firing, kicking, pushing, sticking, weapon, and burning among the reproductive age group in the last 12 months was 14.7% (95%CI: 11.7, 17.4), whereas 15% of women of reproductive age were victims of lifetime physical violence (Table 2).

**Table 2:** Physical violence in women of reproductive age in Gedeo zone 2018 (N=580)

Variable		Number	Per cent
Physical violence (any type like slapping, firing, kicking, pushing, sticking, gunshot, and burning) in 12 months	Yes	85	14.7
	No	495	85.3
Lifetime experienced physical violence	Yes	87	15.0
	No	493	85.0
Who performed physical violence(N=85)	Previous partner	15	2.6
	Current husband	50	8.6
	Father	11	1.9
	Brother	9	1.5
Types of physical violence (N=85)	Firing	8	1.4
	Kicking	18	3.1
	Slapping	19	3.3
	Pushing	12	2.1
	Sticking	19	3.3
	By weapon	7	1.2
	Burning	2	0.3
Cause of physical violence (N=85)	Intoxication	26	4.5
	Distrust	20	3.4
	Unwanted pregnancy	17	2.9
	Low income	5	0.9
	He made other crime	6	1
	Lack of social support	4	0.7
	Has psychiatric problem	3	0.5
	Has an addiction to drugs	4	0.7
Had disagreement	Yes	45	7.8
	No	535	92.2
Has habits like alcohol, chat, and cigarette	Yes	34	5.9
	No	546	94.1
The habit of seeing the film, Facebook, and others	Yes	15	2.6
	No	565	97.4
Any habit of the spouse	Yes	165	28.4
	No	415	71.6
Spouse, the habit of seeing the film, Facebook, and others	Yes	53	9.1
	No	527	90.9

**Factors associated with physical violence in women of reproductive age**: After adjusting the confounding variables, spouse habit significantly associated with alcohol drinking, chat chewing, and cigarette smoking and spouse had watched pornography significantly associated with physical violence in women of reproductive age.

Addict spouse had experienced three times physical violence in women of reproductive age than a non-addict spouse (AOR: 3.56; 95%CI: 1.75, 7.25), and a spouse who watched pornography had experienced 58% of physical violence in women of reproductive age than spouse did not watch pornography ((AOR: 1.58; 95%CI: 1.02, 3.17) (Table 3).

**Table 3:** Factors associated with physical violence in women of reproductive age in Gedeo zone 2018(N=580)

Variable	Physical violence			
	
	Yes	No	COR	AOR
Residence				
Rural	41	44	1.66(1.04,2.64)*	1.97(0.33,1.81)
Urban	178	317	1	
Discussion with husband desire number of children				
Yes	42	43	1.79(1.12,2.84)*	0.63(0.10,0.18)
No	175	320	1	
Mobile				
Yes	40	45	1	
No	171	324	1.68(1.06,2.68)*	1.22(0.21,2.02)
Radio				
Yes	41	44	1	
No	179	316	1.65(1.04,2.61)*	0.88(0.22,2.46)
The woman has any habits like alcohol, chat and cigarette				
Yes	64	21	2.17(0.68,6.98)	1.60(0.80,3.21)
No	482	13	1	
The spouse has any habit like drinking alcohol, chat chewing and cigarette smoking				
Yes	63	22	10.77(6.33,18.32)*	3.56(1.75,7.25)*
No	104	391	1	
Spouse sees pornography film				
Yes	18	67	3.53(1.89,6.59)*	1.58(1.02,3.17)*
No	35	460	1	1

## Discussion

Violence against women is a burning issue in both developed and developing countries. Physical violence is one of the pervasive acts of violation of fundamental human rights. The finding showed that physical violence among women of reproductive age in the Gedeo zone was 14.7%. This finding is slightly higher than the study done in India 9.5% (21), in Iran 9.1% (22) in Bangladesh 10.2% (23), in Turkey 8.1% (24), in Soweto South Africa 5.5 % (25), in South Africa 9 % (26), in Ghana 5 % (27), and in Northwest Ethiopia 11.3 % (7). The possible justification could be slightly higher than other studies: the study time, setting, and socioeconomic difference. This finding is similar to the study done in Bangladesh, 12.4% (28), in India, 12.9 % (29) and Iran, 14.1 % (30). Meanwhile, this finding is lower than the study done in Soweto, South Africa, 25.5 % (25), in Ethiopia, 23% (31) and North Ethiopia, 25.5% (32). The possible justification could be slightly lower than other studies: methodological difference. In this study, slapping is a common type of physical violence. This finding has coincided with a study done in India (29), in Soweto, South Africa (25) and North Ethiopia (32).

In this study, the spouse any habit of alcohol drinking, chat chewing, and cigarette smoking is significantly associated with physical violence among women of reproductive age. This finding is similar to the study done in India (29), South Africa (25), Turkey (24), and North Ethiopia (32). In this study, the spouse's habits, like alcohol drinking, khat-chewing, and cigarette smoking, were determinant factors for physical violence. It has been identified that substance use escalating the happening and severity of physical violence among women of reproductive age (33). Any habit like alcohol drinking, chat chewing, and cigarette smoking directly affects psychological and mental functions to lessen self-esteem and control, leaving individuals less capable of negotiating a nonviolent resolution of conflicts within a relationship (34). In this study, the spouse watching pornography was significantly associated with physical violence in women of reproductive age. This finding was similar to a systematic review in low-income countries (35). This study's strength includes relevant variables that were not addressed previously, such as any habit like alcohol drinking, chat chewing and cigarette smoking, and watching pornography. The limitation of this study did not include both sides, such as a spouse. The outcome could be affected by the recall and social desirability biases.

Conclusion: Physical violence among women of reproductive age in the Gedeo zone is higher than in other studies. Spouses had any habit like alcohol drinking, chat chewing, cigarette smoking, and seeing pornography significantly increased physical violence among reproductive-age women. Therefore, the responsible stakeholders should work on the means of the spouse to alleviate any form of habit like alcohol drinking, chat chewing, cigarette smoking and seeing pornography could decrease physical violence in women of reproductive age.

## References

[1] Chhabra M, Fiore LB, Pérez-Villanueva S (2020). Violence Against Women: Representations, Interpretations, and Education. Violence Against Women.

[2] Gill R, Stewart DE (2011). The relevance of gender-sensitive policies and general health indicators to compare the status of South Asian women's health. Women's health issues.

[3] Sen G (2015). Achieve gender equality and empower all women and girls. UN Chronicle.

[4] Assembly G (2015). Sustainable development goals. SDGs Transform Our World.

[5] Ntaganira J, Muula AS, Masaisa F (2008). Intimate partner violence among pregnant women in Rwanda. BMC women's health.

[6] Hillis S, Mercy J, Amobi A (2016). Global prevalence of past-year violence against children: a systematic review and minimum estimates. Pediatrics.

[7] Girmay A, Mariye T, Bahrey D (2019). Intimate partner physical violence and associated factors in reproductive age married women in Aksum Town, Tigray, Ethiopia 2018, and community based study. BMC Res Notes.

[8] Lasong J, Zhang Y, Muyayalo KP (2020). Domestic violence among married women of reproductive age in Zimbabwe: a cross sectional study. BMC Public Health.

[9] Fahmy HH, Abd El-Rahman SI (2008). Determinants and health consequences of domestic violence among women in reproductive age at zagazig district, egypt. J Egypt Public Health Assoc.

[10] Semahegn A, Belachew T, Abdulahi M (2013). Domestic violence and its predictors among married women in reproductive age in Fagitalekoma Woreda, Awi zone, Amhara regional state, North Western Ethiopia. Reprod Health.

[11] El Kady D, Gilbert WM, Xing G (2005). Maternal and neonatal outcomes of assaults during pregnancy. Obstetrics & Gynecology.

[12] Yost NP, Bloom SL, McIntire DD (2005). A prospective observational study of domestic violence during pregnancy. Obstetrics & gynecology.

[13] Coker AL, Sanderson M, Dong B (2004). Partner violence during pregnancy and risk of adverse pregnancy outcomes. Paediatric and perinatal epidemiology.

[14] Lipsky S, Holt VL, Easterling TR (2004). Police-reported intimate partner violence during pregnancy and the risk of antenatal hospitalization. Maternal and Child Health Journal.

[15] García-Moreno C, Pallitto C, Devries K (2013). Global and regional estimates of violence against women: prevalence and health effects of intimate partner violence and non-partner sexual violence.

[16] Ahmed S, Koenig MA, Stephenson R (2006). Effects of domestic violence on perinatal and early-childhood mortality: evidence from north India. American journal of public health.

[17] Boy A, Salihu HM (2004). Intimate partner violence and birth outcomes: a systematic review. International journal of fertility and women's medicine.

[18] Campbell JC, Baty M, Ghandour RM (2008). The intersection of intimate partner violence against women and HIV/AIDS: a review. International journal of injury control and safety promotion.

[19] Dunkle KL, Jewkes RK, Brown HC (2004). Gender-based violence, relationship power, and risk of HIV infection in women attending antenatal clinics in South Africa. lancet.

[20] García-Moreno C, Jansen HA, Ellsberg M (2005). WHO multi-country study on women's health and domestic violence against women.

[21] Salvi P, Pardeshi G, Bhosale R (2014). Magnitude and risk factors for physical domestic violence during pregnancy. International journal of scientific study.

[22] Faramarzi M, Esmaelzadeh S, Mosavi S (2005). Prevalence, maternal complications and birth outcome of physical, sexual and emotional domestic violence during pregnancy. Acta Medica Iranica.

[23] Naved RT, Persson LÅ (2008). Factors associated with physical spousal abuse of women during pregnancy in Bangladesh. International family planning perspectives.

[24] Karaoglu L, Celbis O, Ercan C (2005). Physical, emotional and sexual violence during pregnancy in Malatya, Turkey. The European Journal of Public Health.

[25] Dunkle KL, Jewkes RK, Brown HC (2004). prevalence and patterns of gender-based violence and revictimization among women attending antenatal clinics in Soweto, South Africa. American journal of epidemiology.

[26] Matseke G, Peltzer K, Mlambo G (2012). Partner violence and associated factors among pregnant women in Nkangala district, Mpumalanga. South African Journal of Obstetrics and Gynaecology.

[27] Pool MS, Otupiri E, Owusu-Dabo E (2014). Physical violence during pregnancy and pregnancy outcomes in Ghana. BMC pregnancy and childbirth.

[28] Silverman JG, Gupta J, Decker MR (2007). Intimate partner violence and unwanted pregnancy, miscarriage, induced abortion, and stillbirth among a national sample of Bangladeshi women. BJOG: An International Journal of Obstetrics & Gynaecology.

[29] Peedicayil A, Sadowski LS, Jeyaseelan L (2004). Spousal physical violence against women during pregnancy. BJOG: An International Journal of Obstetrics & Gynaecology.

[30] Abdollahi F, Abhari FR, Charati JY (2014). Impact of psychological violence on pregnancy outcomes in a prospective study. Iranian journal of psychiatry and behavioral sciences.

[31] Csa I (2016). Ethiopia demographic and health survey.

[32] Feseha G, Gerbaba M (2012). Intimate partner physical violence among women in Shimelba refugee camp, northern Ethiopia. BMC public health.

[33] Kaur R, Garg S (2010). Domestic violence against women: A qualitative study in a rural community. Asia Pac J Public Health.

[34] Room R, Babor T, Rehm J (2005). Alcohol and public health. Lancet.

[35] Grose RG, Chen JS, Roof KA (2021). Sexual and Reproductive Health Outcomes of Violence Against Women and Girls in Lower-Income Countries: A Review of Reviews. J Sex Res.

